# Photoluminescence Quenching of a Novel Electroconductive Poly(propylene thiophenoimine)-co-Poly(ethylenedioxy thiophene) Star Copolymer

**DOI:** 10.3390/polym12122894

**Published:** 2020-12-03

**Authors:** Anne Lutgarde Djoumessi Yonkeu, Miranda Mengwi Ndipingwi, Chinwe Ikpo, Kelechi Nwambaekwe, Sodiq Yussuf, Hayelom Tesfay, Emmanuel Iwuoha

**Affiliations:** SensorLab, University of the Western Cape Sensor Laboratories, Robert Sobukwe Road, Bellville 7570, Cape Town, South Africa; 3318577@myuwc.ac.za (M.M.N.); cikpo@uwc.ac.za (C.I.); 3705852@myuwc.ac.za (K.N.); 3773164@myuwc.ac.za (S.Y.); 3779750@myuwc.ac.za (H.T.); eiwuoha@uwc.ac.za (E.I.)

**Keywords:** electron donor, photoinduced charge transfer, photoluminescence quenching, poly(propylene imine) dendrimer, star copolymer

## Abstract

A generation 1 poly(propylene thiophenoimine)-co-poly(ethylenedioxy thiophene) (G1PPT-co-PEDOT) star copolymer, which exhibits a strong optical absorption over a broad range in the ultraviolet–visible (UV-Vis) region and with good electro/conductive properties, was chemically prepared for the first time. Synthesis of the star copolymer, G1PPT-co-PEDOT was confirmed by spectroscopic studies. Indeed, the disappearance of the very high intensity bands, C–H bending at α-position (687 cm^−1^), and C=N stretching (1620 cm^−1^) in the Fourier transform infrared spectroscopy (FTIR) of G1PPT-co-PEDOT, which were initially present in the spectrum of the thiolated starting material, G1PPT, confirmed copolymerization. Furthermore, a large bathochromic shift in the onset wavelength of the UV-Vis absorbance spectra from 367 nm in G1PPT to 674 nm in G1PPT-co-PEDOT further attests of successful copolymerization. The electrochemical analysis of G1PPT-co-PEDOT achieved a highest occupied molecular orbital (HOMO) energy level value of 5.3 eV, which is reminiscent of the value for an ideal electron-donor material. Photoluminescence quenching of up to 82% was observed in solution blends of the G1PPT-co-PEDOT star copolymer and *N*,*N*′-diisopropyl naphthalene diimide (NDI). This demonstrates the occurrence of photoinduced intermolecular charge transfer (PICT) from the electron-donating G1PPT-co-PEDOT to the electron accepting NDI, a good property, beneficial for optoelectronic and photovoltaic applications.

## 1. Introduction

The discovery of electrical conductivity in chemically doped polyacetylene, has prompted a lot of research in the area of modelling, fabrication, and detailed studies of the properties and applications of π-conjugated polymers [1,2]. Conjugated polymers attract more attention today compared to inorganic semiconductors due to their low production cost, ease of processability, and flexibility, as well as the tunability of their optical and electronic properties through chemical modifications. These first-class properties therefore qualify them as advanced materials in the field of photonics and electronics [3,4]. Four main classes of conjugated polymers, classified according to the structure of their conjugated backbone, have been studied for optoelectronic device applications [5,6,7,8,9,10,11,12]. Insertion of side-chains in these polymers decreases backbone rigidity, increases their solubility and thermal stability, and enables the preparation of nanostructured layers through inexpensive, solution-based methods such as spin-coating [13,14]. These ramifications also account for the tunability of the photophysical and electrochemical properties of these polymers using various routes [15,16,17]. Considerable scientific focus has been put on polythiophenes and their derivatives for their superior chemical and physical properties [18]. Among the polythiophene derivatives, poly(ethylene dioxythiophene), PEDOT is one of the most successful conducting polymers because of its low band gap, excellent environmental stability, high electrical conductivity, and transparency on thin oxidized films, which confer PEDOT good properties for applications in organic optoelectronic and photovoltaic applications [19]. Besides straight-chain polymers, there is a category of branch-like conducting materials referred to as dendrimers. They constitute an interesting class of molecules which have also found sound use in organic optoelectronic applications [15,16,17]. Just like polymers, they are built from smaller repeating sub-units, but instead of generating linear chains, the sub-unit branches out in a well-defined pattern from a central point [20,21]. The non-linear and accurately controlled covalent structure of this class of polymers induced a wide range of studies [22]. π-conjugated dendrimers have shown to be efficient charge transporters in organic light-emitting diodes, attributed to the high-quality of the films formed by these dendrimers [23]. Indeed, due to the strong π–π co-facial interactions within these molecules, they exhibit an extremely high degree of molecular ordering. The nanostructured morphology of dendrimers is aided by their monodisperse nature, which provides them a potential advantage over common polymers [24].

On the other hand, more materials with electron-accepting properties are also being investigated in order to replace fullerene and its derivatives. Among them, the class of molecules called rylenes has been the focus of extensive research [25]. In this interesting class of molecules, 1,4,5,8-naphthalene diimides (NDIs) are six-membered aromatic compounds that have found applicability in many fields, particularly in the design of conducting materials [26,27]. Indeed, they have attracted much attraction due to their tendency to form more *n*-type over *p*-type semiconductors [28,29,30]. Additionally, they are known to be compact, electron-deficient, and capable of self-organization [31,32] and they can also be easily incorporated into larger multicomponent assemblies, through intercalations [33]. In addition, NDIs’ planar aromatic structure allows them to exhibit stacking in the solid state with distances commensurate with π–π stabilization. This property finds use when formation of continuous stacks is necessary [34]. As NDIs have desirable electronic and spectroscopic properties over pyromellitic diimides, they appear as key elements for the design and development of supramolecular functional materials [35,36]. Naphthalene diimides great potential as *n*-type (electron-transporting) semiconductors in organic photovoltaics and optoelectronics lies in their compact and electron deficient cores [37,38,39], and also in their photochemical stability, as well as their air and thermal stabilities [40,41].

Dendrimers based on PEDOT have been mostly prepared electrochemically and studied for applications in bio/sensors or in supramolecular chemistry on substrates [42,43]. Herein, we report the first-time chemical synthesis of an ethylenedioxythiophene-based star copolymer, G1PPT-co-PEDOT and its characterization. The ability of the star copolymer to donate electrons to an electron accepting material, notably *N*,*N*′-diisopropyl naphthalene diimide in different ratios of solution blends of both materials, was also investigated. The electron donating properties of the material were ascertained by evaluating its molecular orbital levels via cyclic voltammetry and by studying its propensity to induce photoluminescence quenching when mixed with *N*,*N*′-diisopropyl naphthalene diimide.

## 2. Materials and Methods

### 2.1. Materials and Reagents

Generation 1 poly(propylene imine) tetramine (DAB-Am4) dendrimer (G1PPI); 1,4,5,8-naphthalene tetracarboxylic dianhydride; 2-thiophene carboxaldhyde, 98%; 3,4-ethylenedioxythiophene, ammonium persulfate, 97%; lithium perchlorate anhydrous; chloroform, 99.9% HPLC grade; *N*,*N*-dimethylsulfoxide, analytical grade; *N*,*N*-dimethyl formamide anhydrous, 99.8%; dichloromethane chromasol plus HPLC, ≥99.9%; methanol, analytical grade; and tetrabutyl ammonium hexafluorophosphate, ≥99.0%; were all purchased from Sigma-Aldrich, Johannesburg, South Africa.

### 2.2. Synthetic Routes

#### 2.2.1. Generation 1 Poly(Propylene Imine) Tetramine (G1PPI) Functionalization

**Scheme 1:**
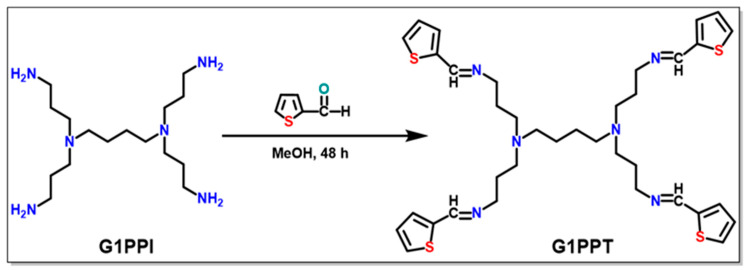
Functionalization of G1PPI to form G1PPT.

0.3863 g (1.2205 mmol) of generation 1 poly (propylene imine) tetramine dendrimer, G1PPI was dissolved in 25 mL of methanol, to which 472.55 µL (4.8821 mmol) of 2-thiophene carbaldehyde was added. The mixture was allowed to stir for 48 h under nitrogen (N_2_) gas [42]. Upon completion of reaction, the solvent was evaporated and washed consecutively with an excess mixture of dichloromethane and water, and G1PPT (see structure in Scheme 1) was collected.

#### 2.2.2. Preparation of G1PPT-co-PEDOT Star Copolymer

**Scheme 2:**
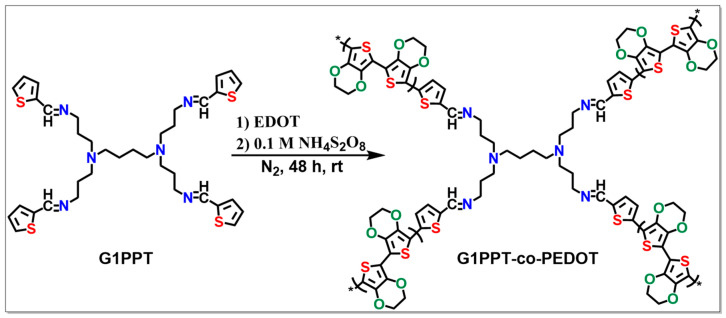
Copolymerization of G1PPT and EDOT to form G1PPT-co-PEDOT.

495 mg (0.71424 mmol) of G1PPT was dissolved in 10 mL of chloroform. Then, 318 µL (2.8570 mmol) of the monomer EDOT was then added with 29.75 mL of 0.1 M (NH_4_)_2_S_2_O_8_ and the mixture was allowed to stir for 2 h at room temperature. Copolymerization is believed to have happened at the interface between the aqueous and the organic phases considering both materials were solely soluble in the respective solvents in which they were prepared. Both solvents were removed by evaporation upon completion of the reaction, which was quenched by addition of methanol. The material was then washed with water and acetone, sonicated, and centrifuged. The star-copolymer G1PPT-co-PEDOT (see structure in Scheme 2) was allowed to dry at ambient air overnight. A solubility test of the prepared polymer was conducted in common organic solvents, and star copolymer was found to only dissolve well in DMSO.

#### 2.2.3. Synthesis of *N*,*N*′-diisopropyl naphthalene diimide (NDI)

**Scheme 3:**
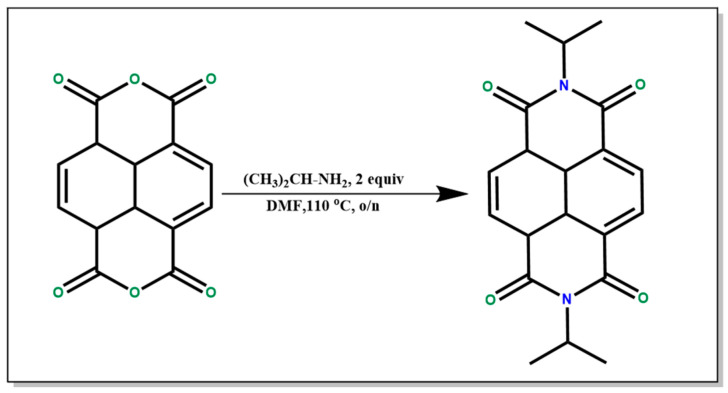
Synthesis of *N*,*N*′-diisopropyl naphthalene diimide (NDI).

2.25 g (8.3900 mmol) of 1,4,5,8- naphthalene tetracarboxylic dianhydride (NTCDA) was introduced in the 2-neck round bottom flask containing 25 mL of *N*,*N*-dimethyl formamide to which two (2) equivalences of isopropylamine (1.42 mL, 0.9919 mol) were added and the mixture was stirred at 110 °C overnight [26]. Filtration of the prepared material, followed by immersion in ice-cold water for 5 min and subsequent filtration, allowed the production of a yellow-canary NDI (see structure in Scheme 3).

### 2.3. Instrumentation

Various analytical techniques were used in order to characterize the prepared materials. Fourier-transform infra-red (FTIR) spectroscopy, used for structural characterization, enabled confirming the presence of the functional groups characterizing the polymeric materials. The analyses were carried out on PerkinElmer model Spectrum 100 series equipment (Shelton, CT, USA). Optical and photo–physical investigation, in the form of ultraviolet–visible (UV-Vis) and photoluminescence (PL) spectroscopy measurements were performed in a DMSO containing quartz cuvette using a Nicolet Evolution 100 UV-Visible spectrometer (Thermo Electron, Pontypool, UK) and a NanoLog^TM^ 3-22-TRIAX spectrofluorometer (Horiba JobinYvon, Palaiseau, France) with double grating excitation, and emission monochromators with a slit width of 5 nm, respectively. Surface morphology and elemental characterization were by scanning electron microscopy (SEM) using a ZEISS ULTRA scanning electron microscope, equipped with an energy dispersive spectrometer (EDS), and a Tecnai G2F2O X-Twin MAT 200 kV field emission transmission electron microscope from Field Electron and Ion Company (FEI), Eindhoven, Netherlands). X-ray diffraction patterns of the material was obtained using a D8 advance diffractometer (BRUKER-AXS, Bruker AXS, GmbH, Karlsruhe, Germany) employing copper Ka radiation with a wavelength of 0.154 nm, operating at a voltage of 40 kV and current of 40 mA. Cyclic voltammetry (CV) studies were conducted on a BAS 100 B electrochemical analyzer from Bioanalytical Systems Incorporated (BASi, West Lafayette, IN, USA). Electrochemical measurements of drop-coated films of G1PPT-co-PEDOT and NDI were performed at ambient air in aqueous solutions of 0.1 M lithium perchlorate (LiClO_4_) and 0.1 M tetrabutylammonium hexafluorophosphate (Bu_4_NPF_6_), respectively. A three-electrode system with gold (Au) and platinum (Pt) working electrodes for G1PPT-co-PEDOT and NDI, respectively, a Pt wire as the counter electrode and Ag/AgCl as reference electrode, was used for these analyses. The sweeping scan rate used was 50 mV/s.

### 2.4. Preparation of the Solution Blends, G1PPT-co-PEDOT:NDI

0.01 M of G1PPT-co-PEDOT was prepared by dissolving 0.06930 g of the star-copolymer in 10 mL of DMSO and sonicated for 1 h. Meanwhile, 0.01 M of NDI was also prepared by dissolving 0.0350 g of NDI in 10 mL of DMSO. Three solution blends of G1PPT-co-PEDOT:NDI were made by mixing one equivalent volume (in mL) of G1PPT-co-PEDOT with different volumes NDI in the ratio 1:1, 1:2, and 1:3, respectively.

## 3. Results and Discussion

### 3.1. Spectroscopic and Morphological Sstudies

**Figure 1:**
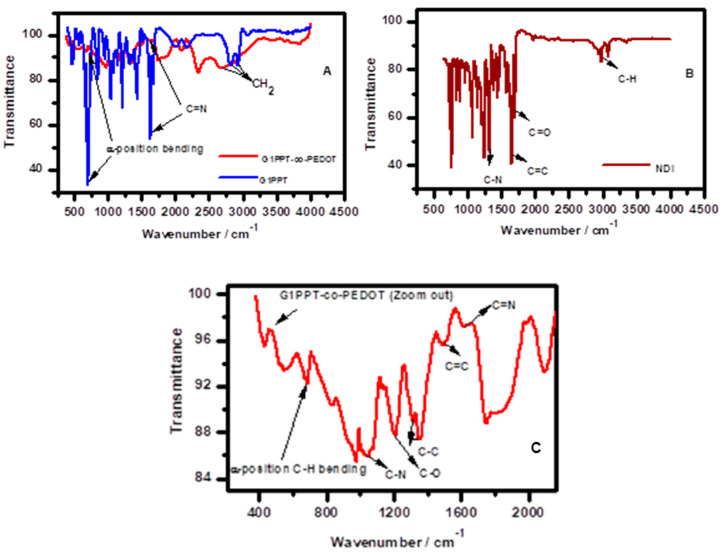
FTIR spectra of G1PPT and G1PPT-co-PEDOT (**A**) and NDI (**B**). Zoomed out spectrum of G1PPT-co-PEDOT (**C**).

Chemically prepared G1PPT, G1PPT-co-PEDOT, and NDI were analyzed by Fourier transform infrared spectroscopy (Figure 1) to confirm the presence of the functional groups inherent to each prepared material (summarized in Table 1). Infrared (IR) spectrum of G1PPT (Figure 1A) showed several characteristic peaks at 475, 705, 833, 845, 1037, 1075, 1217, 1319, 1435, 1629, 1679, 2830, 2938 cm^−1^. Out-of-plane bending of the C–H bond located at the α-position to the thiophene ring was observed at 705 cm^−1^. At 1435 cm^−1^, the medium-weak multiple band accounts for the C=C stretching in the 5-membered aromatic ring of thiophene. The sharp band at 1632 cm^−1^ stretching is attributed to the C=N bond, formed during the functionalization of G1PPI. The bands at 2830 and 2938 cm^−1^ in G1PPT indicate the presence of the –CH_2_ stretching in the dendrimer moiety. In the G1PPT-co-PEDOT spectrum (Figure 1, G1PPT-co-PEDOT zoom), the band of C=N now appears at 1620 cm^−1^; this shift to a lower wavenumber is in agreement with spectroscopic results of electrochemically prepared G1PPT-co-PEDOT reported in literature [42]. A shift of the C–H bending at α-position was also observed from 705 cm^−1^ in G1PPT, to 687 cm^−1^ in G1PPT-co- PEDOT due to the effect of conjugation [44]. Bands at 1548, 1350, and 1320 cm^−1^ are for the conjugated C=C and C–C stretching in the thiophene ring. The bands at 1212 and 1054 can be assigned to stretching modes of ethylenedioxy group. Finally, at 975, 820, and 678 are the vibration modes of C–S bond in the thiophene ring [45]. On the other hand, NDI presents characteristic bands at 1325, 1653, 1698, 2932, 2978, and 3084 cm^−1^ of which the bands at 2932 and 2978 cm^−1^ correspond to the C–H stretching in the isopropyl groups, confirming the imidization of NTCDA. While bands at 1698, 1653, and 1325 cm^−1^ are due to C=O, C=C, and C–N [34].

**Figure 2:**
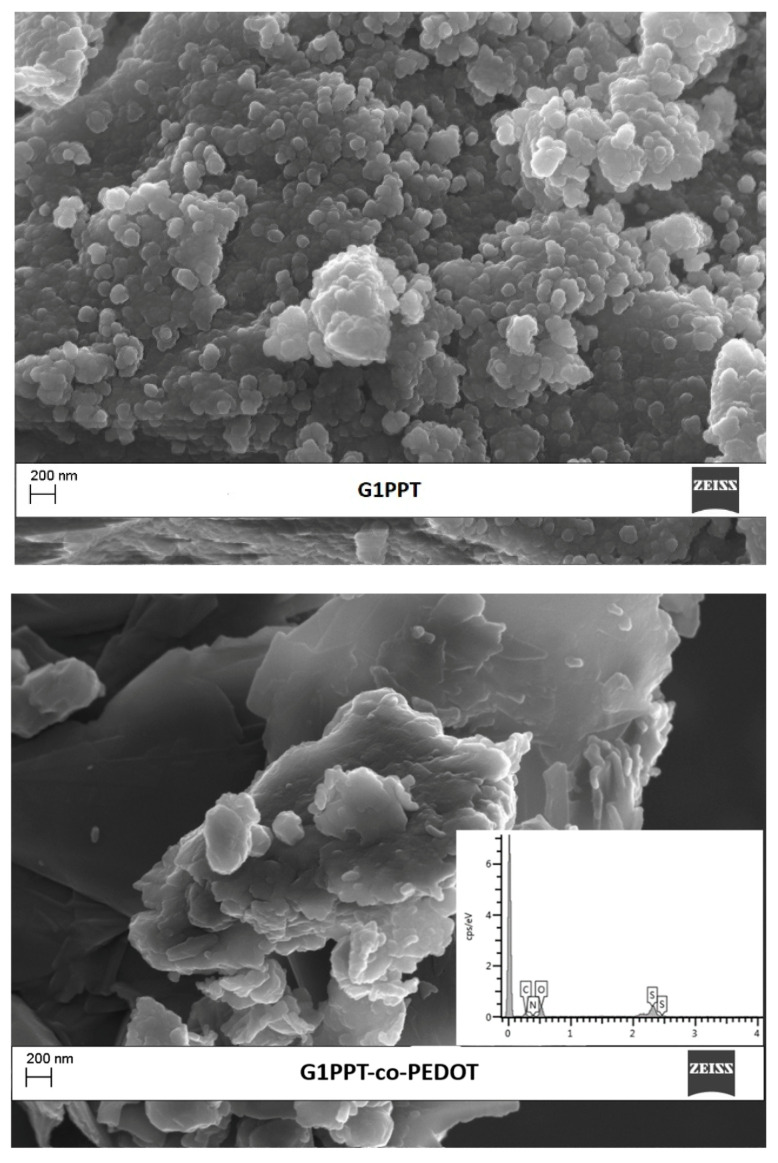

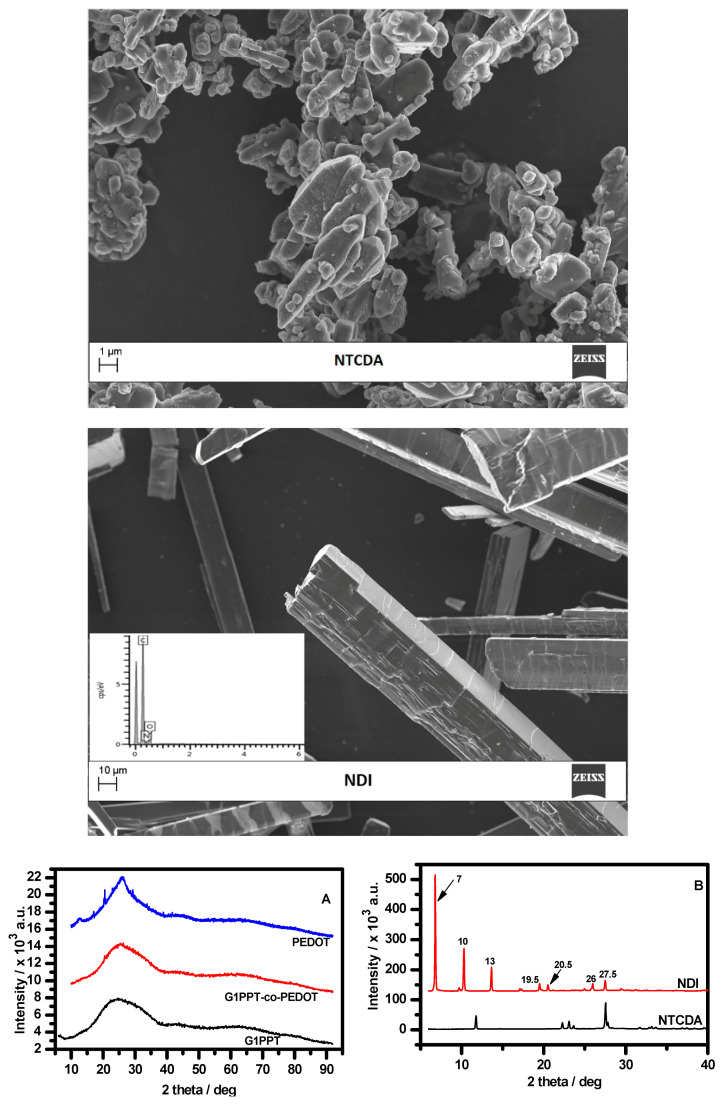
SEM images of G1PPT, G1PPT-co-PEDOT (with its Energy Dispersive X-Ray spectrum insert), NTCDA and NDI (with its Energy Dispersive X-Ray spectrum insert). Graphs: XRD spectra of (**A**) PEDOT, G1PPT-co-PEDOT and G1PPT; and (**B**) NDI and NTCDA.

Structural morphology of G1PPT-co-PEDOT, NDI and their respective starting materials in the form of electron micrograph images obtained from SEM are found in Figure 2. While G1PPT is characterized by an aggregation of closely packed globular molecules, G1PPT-co-PEDOT images show some amorphous, flaky waxy structure at the nanometer level, very similar to the reported morphology of chemically prepared poly(propylene imine) dendrimer-polypyrrole [46]. Such morphology is different from the reported morphology of electro-polymerized G1PPT-co-PEDOT, with mushroom-like structure in which the PEDOT dendrons grow out from the G1PPT moiety core [47]. The observed difference in morphology is suggested to be as a result of possible cross-linking that forces the molecule to remain planar. On the other hand, contrarily to NTCDA which does not present any specific shape or morphology, NDI demonstrates highly well-defined rectangular stick-like shapes in the micrometers range. In addition, the elemental composition of G1PPT-co-PEDOT and NDI were investigated by means of energy dispersive spectroscopy (EDS), and are presented as graphs inset in the materials SEM images. As observed, all expected elements are present in each material, respectively.

X-ray diffraction (XRD) was used to investigate the crystal structure and/or phase purity of the star copolymer, G1PPT-co-PEDOT, and small electron accepting material, NDI. Graphs A and B in Figure 2 below depict the XRD pattern of these materials taken at a 2θ range of 5–40. While G1PPT-co-PEDOT and G1PPT are amorphous in nature as already observed on SEM images, NDI present many peaks, with the most intense at 7 deg. The sharp and intense peaks indicate the crystalline nature of the material [48]. The XRD profile of NDI is almost similar to the diffraction pattern of some fluorinated naphthalene diimides compounds, reported by Katz et al. [49].

### 3.2. Electrochemical Studies

**Figure 3:**
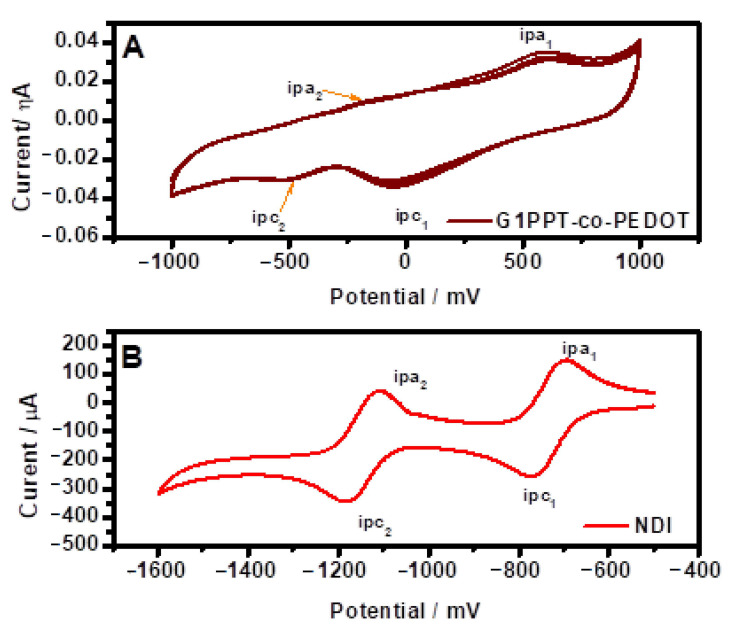
Cyclic voltammograms of G1PPT-co-PEDOT (**A**) and NDI (**B**).

**Scheme 4:**
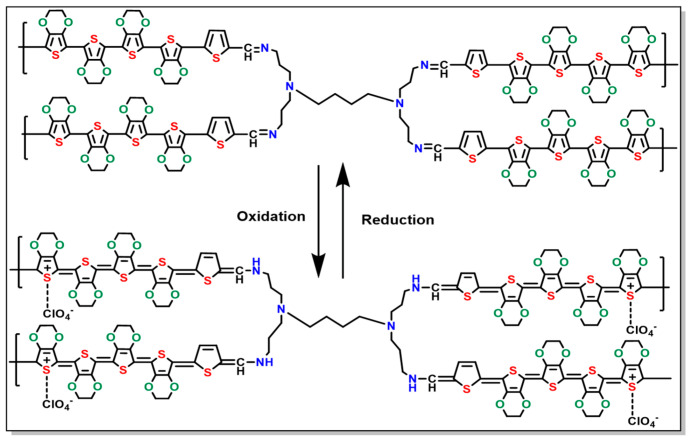
Redox mechanism of G1PPT-co-PEDOT.

The electrochemical behaviors of the polymer G1PPT-co-PEDOT and small molecule, NDI were investigated by cyclic voltammetry (CV) (Figure 3). CV scans were performed in aqueous solutions of 0.1 M LiClO_4_^−^ for G1PPT-co-PEDOT, and 0.1 M Bu_4_NPF_6_ for NDI at a scan rate of 50 mV/s with gold (Au) and platinum (Pt) electrodes used as working electrodes for G1PPT-co-PEDOT and NDI, respectively. The materials were drop-coated on the electrodes. A Pt wire served as the counter electrode and an Ag/AgCl electrode was used as the reference electrode. Figure 3A shows the cyclic voltammogram of chemically prepared G1PPT-co-PEDOT drop-coated on an Au electrode. The graph reveals two distinct redox couples: two oxidation peaks *i_pa1_* and *i_pa2_* at 595 mV and −148 mV and two reduction peaks *i_pc1_* and *i_pc2_* at −51 mV and −503 mV. The persistence of those peaks when sweeping over the potential range at 50 mV/s during five cycles therefore confirmed the obtained peaks. The redox process in the copolymer (see Scheme 4), is as a result of ion diffusion in and out of the film corresponding to the insertion and removal of an ion in the electrolyte [42]. The anodic and cathodic waves could therefore be explained by incorporation of a counter ion. The ClO_4_^-^ anion penetrates into the copolymer matrix and interacts with the oxidation site of the polymer as counter ion. The first redox couple (*i_pa1_* and *i_pc1_*) can be attributed to the introduction and release of the Li^+^ cation while the other redox couple (*i_pa2_* and *i_pc2_*) is associated with the insertion and release of the ClO_4_^−^ anions, respectively [42]. Voltammetric investigation of NDI (Figure 3B) confirmed that this material undergoes two distinct one-electron transfer processes. Indeed, the scan in 0.1 M Bu_4_NPF_6_ prepared in dichloromethane, within a potential range of (−1600)–(−500) mV, at a scan rate of 50 mV/s, revealed two distinct redox couples: two oxidations *i_pa1_* and *i_pa2_* at −700 mV and −1110 mV, and two reduction peaks *i_pc1_* and *i_pc2_* at −768 mV and −1182 mV. This can be explained by a first reduction from NDI to NDI^−^, and a second one from NDI^−^ to the dianion NDI^2−^, giving rise to the two redox couples NDI/NDI^−^ and NDI^−^/NDI^2−^ [26].

Using the linear correlation between the ionization potential energy, *E*_IP_, and oxidation potential and the electron affinity, *E*_A_, and reduction potential derived by Bredas et al. [50] the electrochemical band gap, $E_{g}^{ec}$ of the donor G1PPT-co-PEDOT and NDI were calculated using the following correlations (Equations (1)–(3)) given as:

(1) $$E_{A,LUMO}\;=\;\left|-\left(E_{onset}^{red}+4.4\right)\right|\mathrm{eV}$$

(2) $$E_{1P,HOMO}\;=\;\left|-\left(E_{onset}^{Ox}+4.4\right)\right|\mathrm{eV}$$

(3) $$\;E_{g}^{ec}=\;E_{1P}-\;E_{A}$$

Based on the data from the voltammetric analyses of G1PPT-co-PEDOT, the HOMO and LUMO and energy band gap, $E_{g}^{ec}$ of the donor and acceptor materials were determined and are summarized in Table 2. *E_(HOMO)_*, *E_(LUMO)_*, and $E_{g}^{ec}$ are −5.3 eV, −3.7 eV, and 1.6 eV for G1PPT-co-PEDOT, respectively. For NDI, only the LUMO energy level was determined to be −3.78 eV by cyclic voltammetry, based on the first reduction, which falls within the reported values range of naphthalene diimide based materials [51]. Since in an ideal polymer-based photovoltaic device, the HOMO is determined by the HOMO of the donor, the results obtained reveal that the G1PPT HOMO energy level is very close to the HOMO of an ideal conducting polymer (5.4 eV) [52], confirming its high capability as an electron donating material.

### 3.3. Optical and Photophysical Analyses of G1PPT-co-PEDOT, NDI, and Their Blends

**Figure 4:**
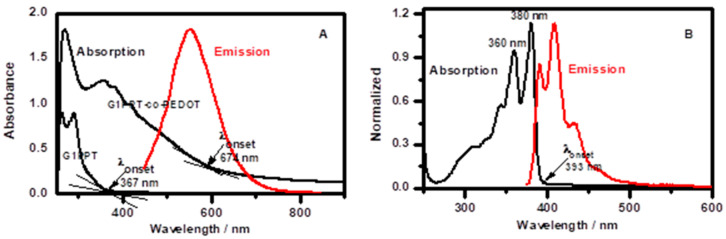
UV-Vis absorption (in black) and photoluminescence emission (in red) spectra of G1PPT and G1PPT-co-PEDOT (**A**) and NDI (**B**).

The optical and photophysical results of the investigated materials were obtained in dimethylsulfoxide (DMSO) as depicted in Figure 4. G1PPT was characterized by two absorption peaks at 263 nm and 281 nm, with approximately the same intensity, and an optical band gap energy,$\;E_{g}^{opt}$ of 3.38 eV. When copolymerized to EDOT, the obtained star copolymer, G1PPT-co-PEDOT demonstrated two peaks, with a maximal absorption intensity at *λ*_max_ *=* 269 nm, whose area under the curve overlaps the two absorption peaks present in G1PPT. This maximal absorption peak is assigned to the absorption by the chromophores within the thiophene moiety. Indeed, according to the literature, reported UV-Vis absorption for thiophene-functionalized dendrimer is between 250–400 nm [47]. One important optical feature of G1PPT-co-PEDOT is the appearance of the broad absorption band that crosses into the visible region between 317 and 700 nm with a maximum absorbance at 355 nm. Such a result confirms the copolymerization, and the presence of PEDOT in the prepared macromolecule, based on the report of UV-Vis characteristics of PEDOT by Hohnholz et al. [53]. The broad absorption is believed to be because of the intramolecular charge transfer, caused by the electronic and polymeric conjugation in the dendritic star copolymer formed [54,55]. With an onset wavelength at 674 nm, the optical band gap is reduced to 1.84 eV compared to 3.38 eV in G1PPT. The presence of this broad, Vis–NIR region absorption band in G1PPT-co-PEDOT shows it could be a good electron donor, with the potential of contributing efficiently to light harvesting in organic optoelectronic and photovoltaic applications. NDI on the other hand was characterized by two major peaks at 360 nm and *λ*_max_ = 380 nm; characteristics of the absorbance of the chromophores C=O and C=C, whose peaks are as a result of π−π^∗^ transitions, which in is agreement with the already reported absorption of NDI [26]. Moreover, the small peak at 344 nm is as a result of *n*–σ* transition within the C–N chromophore. The optical band gap of NDI was determined to be 3.16 eV, reflecting the small conjugated core [56].

The photoluminescence spectra of pristine G1PPT-co-PEDOT and NDI when excited at their respective absorption wavelengths are also shown in Figure 4. Excitation of G1PPT-co-PEDOT at 480 nm gives rise to an emission at 552 nm which corresponds to the relapse of one or more electron(s) from excited state back to its original ground state. Similarly, NDI was excited at 360 nm and three (3) distinct emission peaks at 390 nm, 408 nm, and 433 nm were observed [26]. The Stokes’ shifts, determined as the difference between the peak maxima of absorption and emission, were found to be 282 nm and 28 nm in G1PT-co-PEDOT and NDI, respectively.

**Figure 5:**
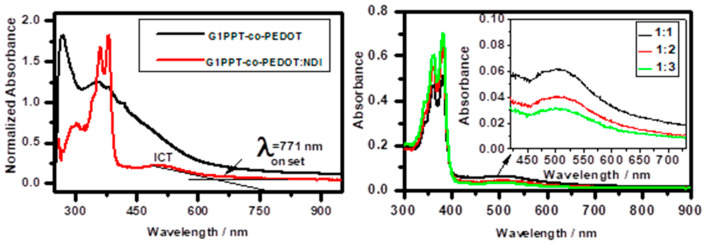
UV-Vis spectra of G1PPT-co-PEDOT:NDI blends, with respect to pristine G1PPT-co-PEDOT.

UV-Vis spectroscopic analysis of G1PPT-co-PEDOT:NDI solution blends was conducted with reference to pristine G1PPT-co-PEDOT (Figure 5), to investigate the donating capability of G1PPT-co-PEDOT. The absorption spectrum of G1PPT-co-PEDOT:NDI solution blend is characterized by the superposition of the absorption bands of G1PPT-co-PEDOT and NDI. Addition of NDI to G1PPT-co PEDOT results in the appearance of a new, clearly defined broad peak between 452–575 nm, with a maximal absorption at longer wavelength at approximately 512 nm, and an absorption onset red-shifted to 771 nm compared to 674 nm in pristine G1PPT-co-PEDOT due to the intermolecular charge transfer between the electron donating star copolymer and the electron accepting NDI [57,58]. Indeed, the highly unsaturated naphthalene diimides core make them good electron accepting materials; and when mixed with G1PPT-co-PEDOT, electrons diffuse from the donating copolymer to NDI.

### 3.4. Photoluminescence Quenching

**Figure 6:**
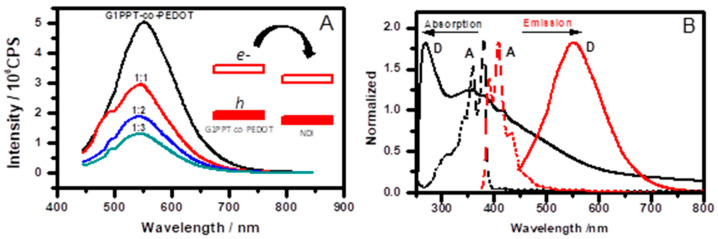
Photoluminescence quenching of G1PPT-co-PEDOT by NDI (**A**). The ratios indicated in (**A**) are for G1PPT-co-PEDOT:NDI. Graph (**B**) represents the absorption (in black) and emission (in red) spectra of G1PPT-co-PEDOT (solid line) and NDI (dashed line), showing no evidence of fluorescence resonance energy transfer. (D: electron donor, G1PPT-co-PEDOT and A: electron acceptor, NDI).

Among other characteristics, polymeric nanocomposites are usually required to exhibit high photoluminescence quenching in order to meet the need for optoelectronic and/or photovoltaic applications [59]. The photoluminescence spectra of pristine G1PPT-co-PEDOT and G1PPT-co-PEDOT: NDI organic solution blends, when excited at a wavelength of 480 nm, are shown in Figure 6A. Photoluminescence quenching is used as an indicator of how well excitons diffusion to a donor-acceptor interface and splitting into free charges take place, and is very beneficial for optoelectronics and photovoltaic applications [60]. It is governed by photoinduced charge transfer and/or resonance energy transfer between species in the excited and ground states, respectively. Energy transfer, on the other hand depends on intermolecular distance, and the overlap of the absorption spectra of the acceptor and emission spectra of the donor [61,62]. When G1PPT-co-PEDOT was excited at 480 nm, an emission at 552 nm was observed with a very intense peak of ca. 5 × 10^6^ CPS. Upon addition of one equivalent volume of NDI, prepared at the same concentration (0.01 M), and excitation at the same wavelength, solution blend of G1PPT-co-PEDOT and NDI (1:1) exhibited approximately 35% fluorescence quenching, characterized by a decrease in intensity to around 3 × 10^6^ CPS. The quenching in G1PPT-co-PEDOT:NDI blend is believed to be due to photoinduced electron transfer [63]. Indeed, considering the energy level alignments of G1PPT-co-PEDOT and NDI (see the inset of Figure 6A), we attribute the fluorescence quenching in G1PPT-co-PEDOT:NDI blends to the electron transfer from G1PPT-co-PEDOT to NDI, with the LUMO difference of 0.70 eV [64]. Moreover, slight blue shifts of the photoluminescence peaks of G1PPT-co-PEDOT:NDI blends, with respect to pristine G1PPT-co-PEDOT, were observed. This could be explained by a potential decrease in density of state at the top of the valence band which could result in an increase in effective band gaps [59]. It is also believed that fluorescence resonance energy transfer does not contribute to the observed quenching, since NDI optical absorption spectrum does not overlap with the G1PPT-co-PEDOT emission spectrum [65], as observed in Figure 6B. Further increase in NDI blend’s content (G1PPT-co-PEDOT:NDI ratio as 1:2, 1:3), results in an increased quenching up to 82%. This was characterized by a further decrease in photoluminescence intensity to 1.9 × 10^6^ CPS and 1.3 × 10^6^ CPS, the in 1:2 and 1:3 solution blends, respectively. Two main types of PL quenching phenomena have been reported to occur in polymers, namely static and dynamic quenching. An interaction between a fluorophore (G1PPT-co-PEDOT) and a quencher (NDI) remains the pillar of this process. When a non-fluorescent complex is formed between a fluorophore and a quencher, regardless of the population of the excited state, a static quenching occurs. On the other hand, diffusion of the quencher to the fluorophore during the lifetime of the excited state results in the occurrence of a dynamic or collisional quenching. Such diffusion will cause the fluorophore to return to the ground state without photon emission. The collisional quenching is optimally described by the Stern–Volmer law [60]. Further transient absorption spectroscopy study is required to elucidate which of these processes occur.

## 4. Conclusios

Novel G1PPT-co-PEDOT star copolymer and its blends with *N*,*N*′-diisopropyl naphthalene diimide (NDI) were synthesized and evaluated for their potential application in optoelectronics as electron donating polymers. The UV-Vis absorption spectra of the star copolymer revealed a broad absorption band in the visible-near infrared (Vis-NIR) region, with an optical band gap as low as 1.84 eV, and a red-shift to higher wavelength in its solution blend with NDI. Effective photoinduced charge transfer was confirmed by photoluminescence quenching of up to 82%, when increasing amount of NDI was added to a solution of pristine G1PPT-co-PEDOT. The content of NDI in the solution blend played a significant role in the photoluminescence (PL) quenching. High NDI contents resulted in improved PL quenching. Characteristics such as high Vis-NIR region absorption, HOMO energy level below oxygen HOMO level (−5.3 eV), propensity to effectively donate electrons to electron accepting materials, and band gap below 2.0 eV (as found in G1PPT-co-PEDOT) are of primary importance for electron donating materials in optoelectronics. Thus, the blending of electroconductive G1PPT-co-PEDOT star copolymer with air-stable NDI may afford optoelectronic and photovoltaic devices with desirable properties, and hence holds promise for such applications.

## Figures and Tables

**Table 1:** Summary of vibrational modes of G1PPT-co-PEDOT and NDI.

Materials	Wavenumber (cm^−1^)	Vibrational Mode
G1PPT-co-PEDOT	687	α-position C–H bending
	1212	C–O stretching
	1310, 1357	C–C stretching
	1495	C=C stretching
	1620	C=N stretching
	2842	C–H stretching
NDI	1325	C–N stretching
	1653	C=C stretching
	1698	C=O stretching
	2932, 2978 3084	C–H stretching (isopropyl group) C–H stretching (aromatic)

**Table 2:** HOMO, LUMO, and band gap $E_{g}^{ec}$ energy levels of G1PPT-co-PEDOT and NDI.

Materials	$E_{onset}^{ox1}$ (V)	$E_{onset}^{red1}$ (V)	*E_(HOMO)_* (eV)	*E_(LUMO)_* (eV)	$E_{g}^{ec}$ (eV)
G1PPT-co-PEDOT	0.87	−0.69	5.3	3.7	1.6
NDI	−0.50	−0.62	-	3.78	-
